# Diffuse Large B-Cell Lymphoma at the Site of a Herpes Zoster Scar

**DOI:** 10.4021/wjon531w

**Published:** 2012-08-26

**Authors:** Rana M. Mays, Rajini K. Murthy, Rachel A. Gordon, Whitney J. Lapolla, Sarah K. Galfione, Amy A. Hassan, Ronald P. Rapini, Carolyn A. Bangert, Stephen K. Tyring

**Affiliations:** a Center for Clinical Studies, Webster, Texas; 451 N. Texas Avenue Webster, TX 77598, USA; b School of Medicine, University of Alabama at Birmingham, Birmingham, Alabama; 1530 3rd Avenue South Birmingham, AL 35294, USA; c Dept of Dermatology, University of Texas Medical School at Houston, Houston, Texas; 6655 Travis Street, Suite 980 Houston, Texas 77030, USA; d Dept of Hematology and Oncology, MD Anderson Cancer Center, Houston, Texas; 1515 Holcombe Blvd Houston, TX 77030, USA; e Depts of Dermatology, MD Anderson Cancer Center and University of Texas Medical School at Houston, Houston, Texas; 6655 Travis Street, Suite 980 Houston, Texas 77030, USA

**Keywords:** Herpes zoster, Varicella zoster virus, Diffuse large B-cell lymphoma

## Abstract

Herpes zoster, also known as shingles, occurs upon reactivation of a primary infection with varicella zoster virus (VZV). Risk factors for reactivation include stress, older age, and immunosuppression, all of which are associated with a decrease in host immunity. Common complications of herpes zoster include scarring and post-herpetic neuralgia (PHN). Cutaneous lesions such as granuloma annulare, lymphomas, and sarcoid granulomas have also been reported to potentially arise at the site of herpes zoster. Here, we report a case that to our knowledge is the first presentation of diffuse large B-cell lymphoma with its only cutaneous manifestation arising in a herpes zoster scar. Punch biopsy was performed on a nodule appearing in a dermatomal distribution within the herpes zoster scar. Histopathology revealed an atypical lymphoid infiltrate in the dermis that was determined to be CD20 positive B-cells. Immunostains for CD20, CD79a, and PAX-5 showed strong positive staining of the atypical cells, confirming B-cell origin and resulting in the diagnosis of lymphoma, large B-cell type. This case highlights the importance of raising clinical suspicion for a malignant process in patients who present with a changing or unresolving skin manifestation after infection with varicella zoster virus.

## Introduction

Infection with varicella zoster virus (VZV), more commonly known as shingles, occurs upon reactivation of a primary varicella infection (chickenpox). After vaccination or primary infection with VZV, the virus enters a dormant state within the dorsal root ganglion. Reactivation, which involves replication of the virus within the dorsal root ganglion of the affected dermatome, is accompanied by the prodromal symptom of pain caused by inflammation and necrosis of neuronal and non-neuronal cells. The virus travels to the skin via the axons of spinal sensory nerves causing an eruption, which classically appears as erythematous macules and papules that progress to grouped vesicles in a unilateral dermatomal distribution. Decreased host immunity is suspected to be the cause of VZV reactivation. Proposed factors of decreased immunity include, but are not limited to, increased stress, older age, malignancy, human immunodeficiency virus infection, and treatment with immunosuppressive agents [1]. A family history of shingles has also been shown to increase the risk of shingles [2]. Complications of infection with VZV include scarring and post-herpetic neuralgia (PHN). PHN is an important public health concern as patients can suffer from extreme pain in the distribution of past infection with VZV that can last from months to years [1].

Diffuse large B-cell lymphomas are classified as a type of non-Hodgkin lymphoma (NHL) exhibiting a diffuse proliferation of large cells with a moderate amount of cytoplasm containing cleaved or non-cleaved nuclei with multiple nucleoli. Immunophenotyping reveals expression of pan-B-cell antigens including CD19, CD20, and CD22. Diffuse large B-cell lymphomas account for 30% of all NHLs. 40% of patients are diagnosed with stage IV disseminated disease, with involvement on both sides of the diaphragm. Stage I or II disease, defined as confinement to one side of the diaphragm, is seen in 30 to 40% of patients. Stage I or IE localized disease is seen in 20% of patients [3].

Primary cutaneous lymphomas are defined as the presence of lymphoma in the skin with no disease found at extracutaneous sites at the time of diagnosis [4]. Primary cutaneous B-cell lymphomas represent about 20-25% of all primary lymphomas making them much less common than primary T-cell lymphomas. Over the past 20 years, it has become clear that some B-cell lymphoma subtypes can present exclusively as skin lesions [5]. At the time of diagnosis made in this case report, extensive extracutaneous involvement was present. Thus, our patient’s case is not considered primary cutaneous B-cell lymphoma, but as cutaneous metastases of large B-cell lymphoma. The diagnosis of cutaneous B-cell lymphoma is established by biopsy, preferably excisional or by punch when possible. Definitive diagnosis is established by immunohistochemical studies. B-cell lineage is confirmed by CD3, CD20, and/or CD79a positivity. The amount of reactive T-cells admixed must also be assessed. The presence of rapidly proliferating cells and the differentiation between neoplastic and reactive follicles is established by Ki-67, which only stains neoplastic and rapidly proliferating cells. Distinction between different types of B-cell lymphoma, pseudo-B-cell lymphoma, and secondary cutaneous B-cell lymphoma is made using Bcl-2, Bcl-6, CD10, MUM-1, and FOXP1 [5].

Here, we report a case that to our knowledge is the first presentation of diffuse large B-cell lymphoma with its only cutaneous manifestation arising in a herpes zoster scar.

## Case Report

A 64-year-old Vietnamese female presented to our dermatology clinic for treatment of post-herpetic neuralgia (PHN) approximately 45 days after diagnosis of herpes zoster infection of the left T-4 dermatome. Upon initial diagnosis of herpes zoster infection, she was treated with acyclovir 800 mg PO 5 times daily for seven days, hydrocodone/acetaminophen 10/350 mg PO q 4-6 hours prn pain, promethazine 25 mg PO q 4 hours prn nausea or vomiting, and gabapentin 600 mg PO daily. At the time of presentation to our clinic, she had completed the course of acyclovir and was taking gabapentin 2400 mg PO daily and hydrocodone/acetaminophen 10/325 mg PO q 4-6 hours prn pain for treatment of post-herpetic neuralgia. Additional medications included vitamin D, calcium supplement, atorvastatin, ibandronate, and teriparatide. Her past medical history was significant for osteoporosis, hyperlipidemia, and hepatitis B. Allergies included penicillin, which caused a macular and papular rash when taken. Family history was significant for breast cancer in two maternal aunts and liver cancer in her maternal uncle. She denied use of tobacco, alcohol and illicit substances. She was a retired accountant and had no known chemical exposures. Review of systems was negative.

On physical examination, she had dermatomal hyperpigmentation and erythema in the distribution of the left T4 dermatome consistent with healing herpes zoster lesions. No lymphadenopathy was appreciated. Upon evaluation at our clinic, her treatment was adjusted to substitute pregabalin for gabapentin and she was instructed to continue to use hydrocodone/acetaminophen prn for pain. Throughout her treatment course, she was tapered off of pregabalin and hydrocodone/acetaminophen, eventually only applying topical lidocaine ointment for pain secondary to post-herpetic neuralgia.

**Figure 1:**
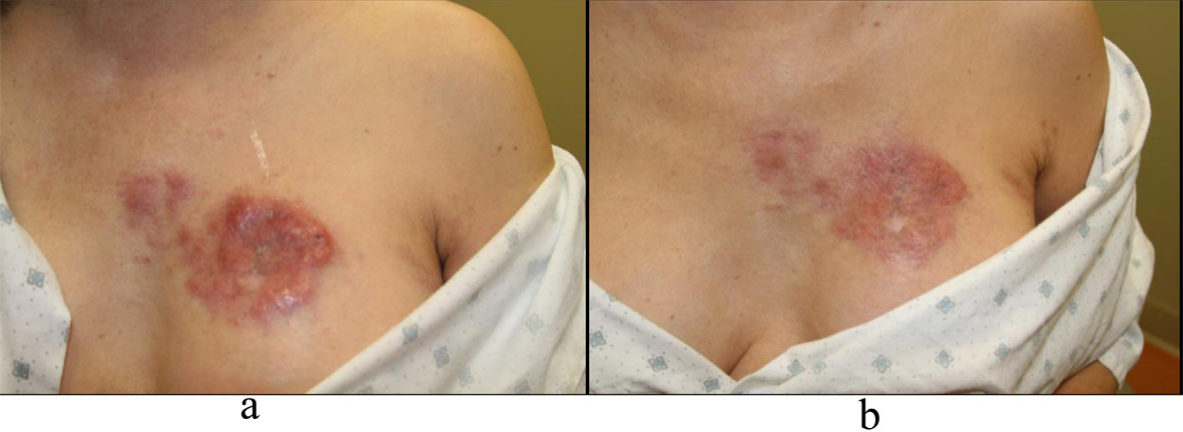
a: Left chest nodules; b: Left chest nodules.

**Figure 2:**
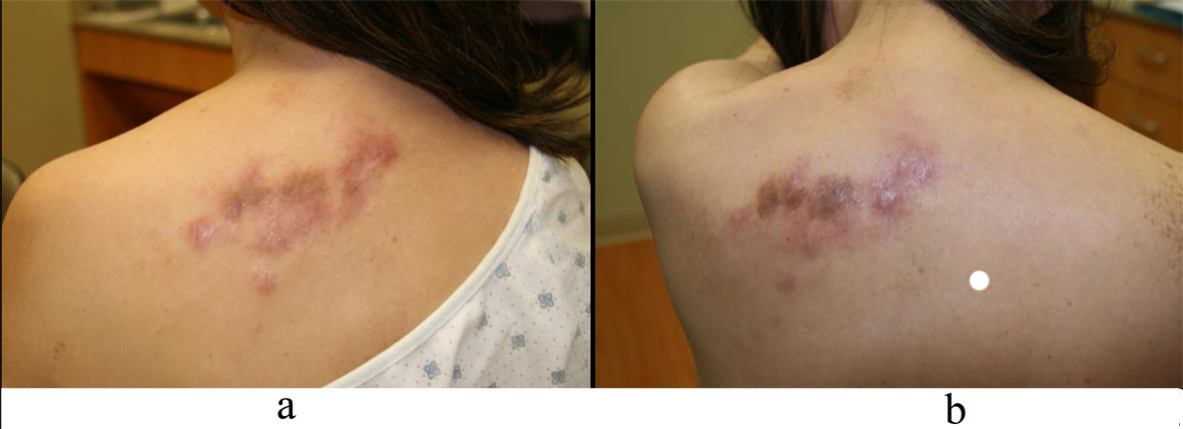
a: Left back nodules; b: Left back nodules.

Four months after diagnosis of shingles, the patient complained of persistence of a scar on her left chest and left back and requested treatment. Physical exam revealed pink to violaceous hypertrophic smooth shiny raised nodules distributed on the left chest (Fig. 1a, b) and left back (Fig. 2a, b). Approximately 10 months after diagnosis of herpes zoster infection, the patient felt the nodules were increasing in size and presented to our clinic for therapy. A 4.0 mm punch biopsy was performed on a nodule of the L chest.

**Figure 3:**
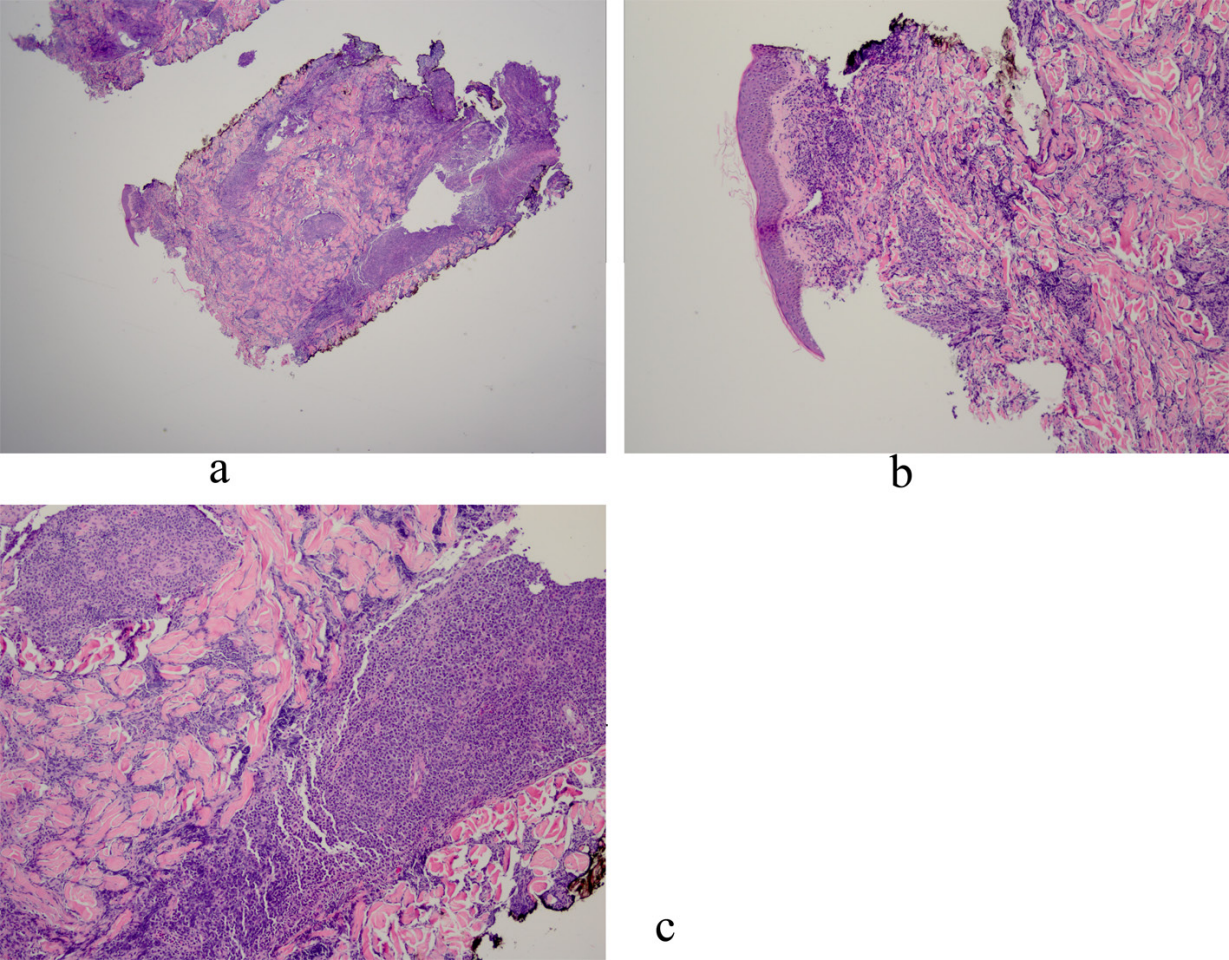
a: Biopsy of left chest nodule reveals atypical lymphoid infiltrate in the dermis with no infiltration into the epidermis; b: Biopsy of left chest nodule reveals atypical lymphoid infiltrate in the dermis with no infiltration into the epidermis; c: Biopsy of left chest nodule reveals atypical lymphoid infiltrate in the dermis with no infiltration into the epidermis.

**Figure 4:**
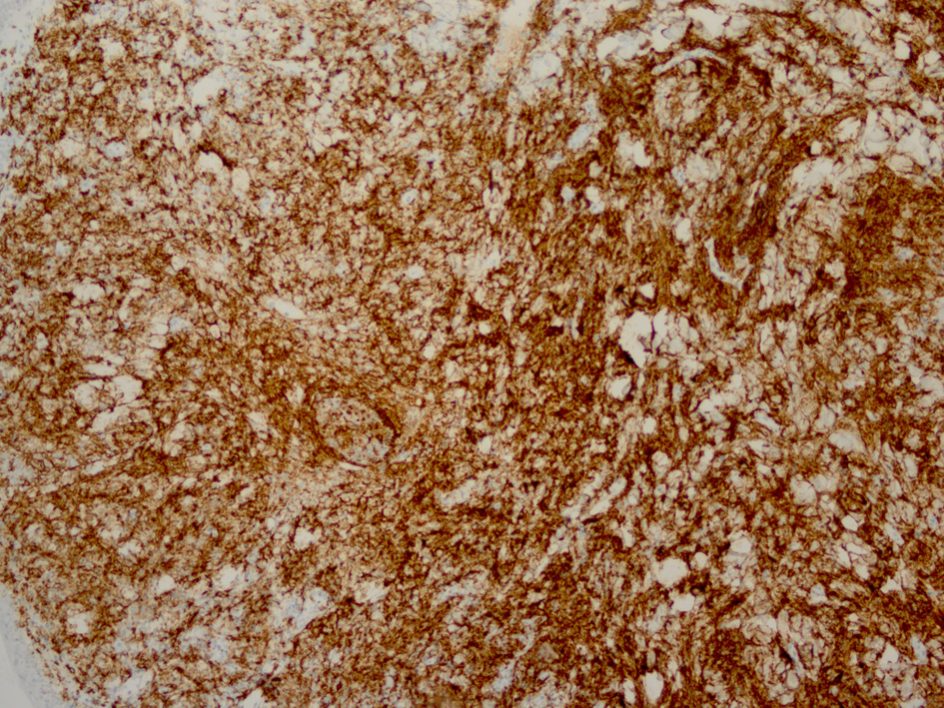
Immunostaining reveals monomorphic atypical lymphocytes diffusely arranged through the dermis as CD20 positive B-cells.

**Figure 5:**
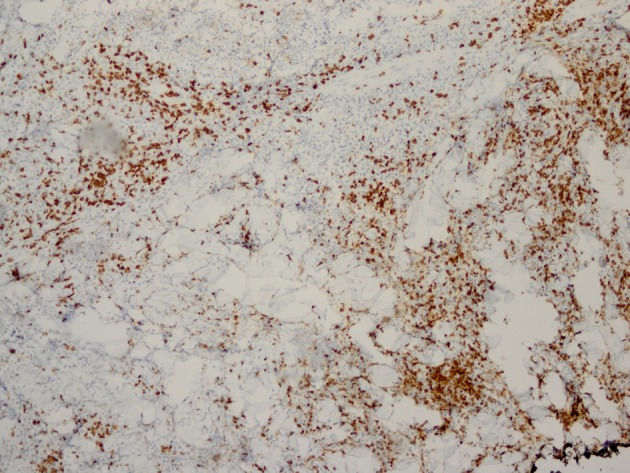
Immunostaining for CD3 reveals smaller non-atypical cells, consistent with reactive T-cells.

**Figure 6:**
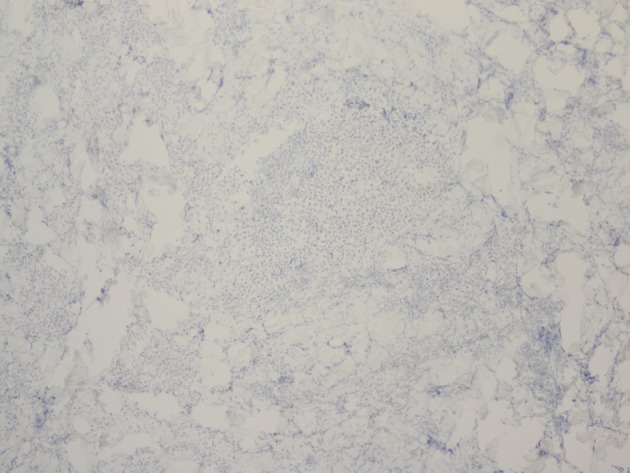
Immunostaining for CD30 reveals few scattered reactive cells.

Histopathology revealed an atypical lymphoid infiltrate in the dermis, but no infiltration into the epidermis (Fig. 3a, b, c). Immunostaining was negative for neuron specific enolase, HMB45, and cytokeratin 20. The majority of infiltrating monomorphic atypical lymphocytes diffusely arranged through the dermis was CD20 positive B-cells (Fig. 4). About 20% of the cells were CD3 positive T-cells. Immunostaining for CD3 showed several smaller non-atypical cells, consistent with reactive T-cells (Fig. 5). Staining for CD30 was positive in only 2% of cells and showed a few scattered reactive cells, but most of the atypical cells did not stain with this marker (Fig. 6). This finding ruled out the possibility of CD30 (Ki-1) lymphoproliferative disorder, such as lymphomatoid papulosis or large cell lymphoma of that spectrum. Immunostains for CD20, CD79a, and PAX-5 showed strong positive staining of the atypical cells, confirming B-cell origin. Immunostaining for TDT was negative, excluding lymphoblastic lymphoma. MUM-1 and Ki-67 showed diffuse staining of the atypical cells. The diagnosis was confirmed as lymphoma, large B-cell type. The patient was referred to an oncologist for further evaluation and treatment.

**Figure 7:**
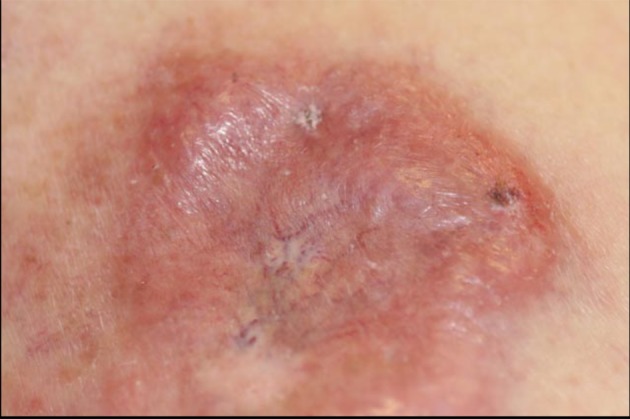
Left breast and inferior left chest wall with skin thickening and nodularity.

She subsequently underwent laboratory evaluation, bone marrow biopsy, and imaging studies. Chemistries and liver function tests were remarkable for a mildly elevated LDH of 673 IU/L (normal = 313 - 618 IU/L), slightly decreased white blood cell count of 3.4 k/mL (normal = 4 - 11 k/mL), and an elevated beta2 microglobulin of 3.7 (normal = 0.7 - 1.8 mg/L). Hepatitis B core antibody was reactive, hepatitis B surface antigen was reactive, and hepatitis C virus antibody was non-reactive. Computerized tomography (CT) scan of the neck, chest, abdomen and pelvis revealed extensive lymphadenopathy, including submandibular, jugulodigastric, spinal accessory, supraclavicular, retroperitoneal and axillary lymphadenopathy. There were multiple bilateral small pulmonary nodules and hilar lymph nodes. There was skin thickening with nodularity in the left breast and inferior left chest wall (Fig. 7). There was bilateral axillary and retroperitoneal lymphadenopathy. There was splenomegaly measuring 13 x 11 x 6.8 cm. Positron emission tomography (PET) scan revealed multi-compartmental FDG-avid adenopathy above and below the diaphragm including a left-sided focus that spanned the majority of the left neck and extended into the supraclavicular region and skin involvement in the head, neck, and thorax. The spleen was enlarged and slightly more avid than the liver, consistent with tumor involvement. There were irregularly marginated pulmonary nodules in the right apex and right upper lobe, which were below resolution of PET, but may represent sequelae of infectious or inflammatory processes. Based on results of imaging studies revealing skin and spleen involvement and disease evident on both sides of the diaphragm, the patient was determined to have stage IV diffuse large B-cell lymphoma. The patient began chemotherapy with cyclophosphamide, hydroxydaunorubicin, vincristine, and prednisone. Given active infection with hepatitis B, the addition of rituximab was held until concomitant treatment with lamivudine was completed.

The patient responded to treatment and returned to clinic one year after completion of chemotherapy. She denied any fever, chills, or increase in lymphadenopathy. CT and PET imaging at the follow-up appointment, however, revealed new and metabolically active lymph nodes concerning for recurrent disease. Interestingly, FNA and core biopsy of the left axillary lymph node revealed low-grade B-cell lymphoma rather than a diffuse large B-cell lymphoma. Further work-up will include excisional lymph node biopsy to confirm the diagnosis of low-grade B-cell lymphoma as well as bone marrow biopsy for staging.

## Discussion

There have been some reported cases of various cutaneous lesions arising at the site of herpes zoster infection. These cutaneous lesions include granuloma annulare, lymphomas, pseudolymphomas, sarcoid granulomas, tuberculoid granulomas, granulomatous vasculitis, Kaposi’s sarcoma, and pyodermatitis vegetans [6, 7]. Unless testing for VZV is performed, it cannot be proven if these lesions arise within a previously infected area or if the skin lesions are appearing in a zosteriform distribution. The pathogenesis of the appearance of neoplasms in a dermatomal distribution following herpes zoster infection is not clearly understood, but several possible mechanisms have been proposed. It has been suggested that infection with herpes zoster decreases local immunity in a dermatomal distribution allowing development of malignant neoplasms or metastases of internal malignancies. This has been proposed as the existence of a “locus minoris resistentiae,” or site of decreased resistance, which attracts neoplastic cells and allows them to proliferate in a dysregulated manner. Another possible mechanism involves a “Koebner-like” phenomenon in which the skin and nerve damaged by herpes zoster infection allows metastases to that area to occur. It is also possible that the inflammation of the spinal sensory nerve allows the metastatic cells to move along in a similar way as the VZV and arrive at the area of VZV skin lesions where they are left to proliferate [6].

There are reported cases of skin lesions previously diagnosed as cutaneous lymphoma later discovered to be pseudolymphoma. This important distinction must be made when evaluating possible cutaneous lymphomas, particularly if preceded by viral infection. Pseudolymphomas mimic cutaneous lymphomas, but are benign proliferations of lymphoid cells. Clinically, pseudolymphomas and lymphomas are indistinguishable. In many cases, histopathology does not clearly establish the diagnosis, requiring immunohistologic or molecular investigations. Pseudolymphomas have also been shown in nodular scabies, persistent nodular arthropod bite reactions, herpes simplex, sites of vaccination, tattoos, infection by *Borrelia burgdorferi*, and in association with molluscum contagiosum [8, 9].

To our knowledge, this is the first case of diffuse large B-cell lymphoma having its only cutaneous presentation solely in a herpes zoster scar. In a search of the recent scientific literature, a few cases of various lymphomas arising in herpes zoster scars have been reported. There is one reported case of leukemia cutis arising in a herpes zoster scar in a patient previously diagnosed with B-cell chronic lymphocytic leukemia [10]. In another reported case, a patient had herpes zoster-like lesions for 2 years and was eventually diagnosed with zosteriform T-cell lymphoma [11]. Additionally, there was one reported case of acute infiltration of B-cell lymphoma in disseminated herpes zoster lesions [12]. Finally, there is one reported case of lymphoplasmocytoid lymphoma and one reported case of centroblastic centrocytic lymphoma arising in a herpes zoster scar [13, 14].

It is important to recognize that the persistence of skin manifestations of herpes zoster infection should raise concern. Nodules and plaques that appear in a dermatomal distribution after infection with herpes zoster may represent something other than a hypertrophic scar. Biopsy should be considered in these patients so that early diagnosis can be made. Further studies are needed to understand the pathophysiology of local immunosuppression in herpes zoster.
